# The spatiotemporal electrogram dispersion ablation targeting rotors is more effective for elderly patients than non‐elderly population

**DOI:** 10.1002/joa3.12860

**Published:** 2023-05-03

**Authors:** Kensuke Sakata, Tomomi Tanaka, Soichiro Yamashita, Masanori Kobayashi, Mitsuaki Ito, Kohei Yamashiro

**Affiliations:** ^1^ Alliance for Cardiovascular Diagnostic and Treatment Innovation Johns Hopkin University Baltimore Maryland USA; ^2^ Heart Rhythm Center Takatsuki General Hospital Takatsuki Japan; ^3^ Department of Cardiology Hyogo Prefectural Awaji Medical Center Sumoto Japan; ^4^ Department of Cardiovascular Medicine Matsumoto Kyoritsu Hospital Matsumoto Japan; ^5^ Department of Cardiovascular Medicine Hyogo Brain and Heart Center Himeji Japan

**Keywords:** atrial fibrillation, catheter ablation, follow‐up study, rotor, spatiotemporal electrogram dispersion

## Abstract

**Background:**

Modulating atrial fibrillation (AF) drivers has been proposed as one of the effective ablation strategies for non‐paroxysmal AF (non‐PAF). However, the optimal non‐PAF ablation strategy is still under debate because the exact mechanisms of AF persistence including focal activity and/or rotational activity, are not well‐understood. Recently, spatiotemporal electrogram dispersion (STED) assumed to indicate rotors in the form of rotational activity is proposed as an effective target for non‐PAF ablation. We aimed to clarify the effectiveness of STED ablation for modulating AF drivers.

**Methods:**

STED ablation plus pulmonary vein isolation was applied in 161 consecutive non‐PAF patients not undergoing previous ablation. STED areas within the entire left and right atria were identified and ablated during AF. After the procedures, the STED ablation's acute and long‐term outcomes were investigated.

**Results:**

(1) Despite a more effective acute outcome of the STED ablation for both AF termination and non‐inducibility of atrial tachyarrhythmias (ATAs), Kaplan–Meier curves showed that the 24‐month freedom ratio from ATAs was 49%, which resulted from the higher recurrence ratio of atrial tachycardia (AT) rather than AF. (2) A multivariate analysis showed that the determinant of ATA recurrences was only a non‐elderly age, not long‐standing persistent AF, and an enlarged left atrium, which were conventionally considered as key factors.

**Conclusions:**

STED ablation targeting rotors was effective in elderly non‐PAF patients. Therefore, the main mechanism of AF persistency and the component of the fibrillatory conduction might vary between elders and non‐elders. However, we should be careful about post‐ablation ATs following substrate modification.

## Graphical abstract

The ablation strategy targeting spatiotemporal electrogram dispersion (STED) suggesting rotors was effective in elderly population. However, more attention must be paid to the post‐ablation atrial tachycardia following substrate modification by the STED ablation.

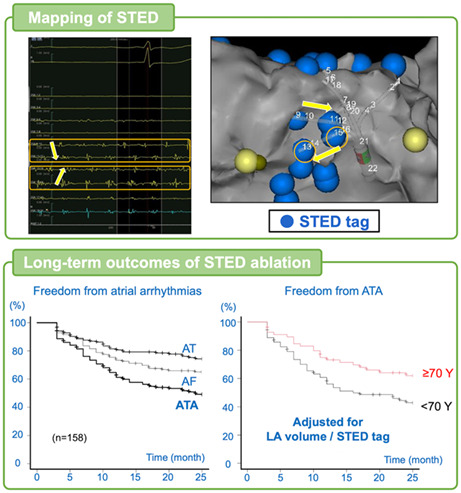

## 1 INTRODUCTION

Catheter ablation via pulmonary vein isolation (PVI) is recognized as an effective treatment for paroxysmal atrial fibrillation (PAF),^1^ and on the other hand, a PVI alone is insufficient for non‐PAF. Therefore, several strategies in addition to the PVI have been performed such as linear ablation and electrogram‐based ablation targeting complex fractionated atrial electrograms (CFAEs).^2^, ^3^ However, previous meta‐analyses have demonstrated that adding these conventional ablation strategies to the PVI is unlikely to have a more superior effect than a PVI alone.^4^, ^5^

In recent years, various systems and strategies for rotor ablation have been reported.^6^, ^7^, ^8^, ^9^ A novel electrogram‐based ablation targeting spatiotemporal electrogram dispersion (STED), assumed to indicate rotors in the form of rotational activity, was reported as one of the effective targets for non‐PAF.^10^ However, few clinical studies have reported the long‐term outcome of STED ablation. This study aimed to clarify the additional efficacy of STED ablation for modulating atrial fibrillation (AF) drivers beyond the conventional ablation strategy.

## 2 METHODS

### 2.1 Study design

One hundred sixty‐one consecutive patients with non‐PAF without previous ablation procedures for AF, who underwent catheter ablation at the Heart Rhythm Center of Takatsuki General Hospital between October 2017 and December 2019, were enrolled in this study. If a thrombus in the left atrium (LA) was detected by enhanced computerized tomography (CT) or transesophageal echocardiography, the procedure was postponed until the thrombus had resolved. Informed consent was obtained from each patient and approved by the ethics committee in our hospital. Antiarrhythmic drugs (AADs), except for amiodarone, were discontinued for at least five half‐lives before the procedure. Oral anticoagulation was stopped for 1 day or half a day on the procedure day to avoid bleeding complications. We infused intravenous heparin after all sheaths were inserted, and controlled the activated clotting time (ACT) every 10–30 min to maintain an ACT of 300–350 s. After placing two transseptal sheaths in the LA via the femoral vein, mapping was started.

### 2.2 Strategy of the STED ablation

**FIGURE 1:**
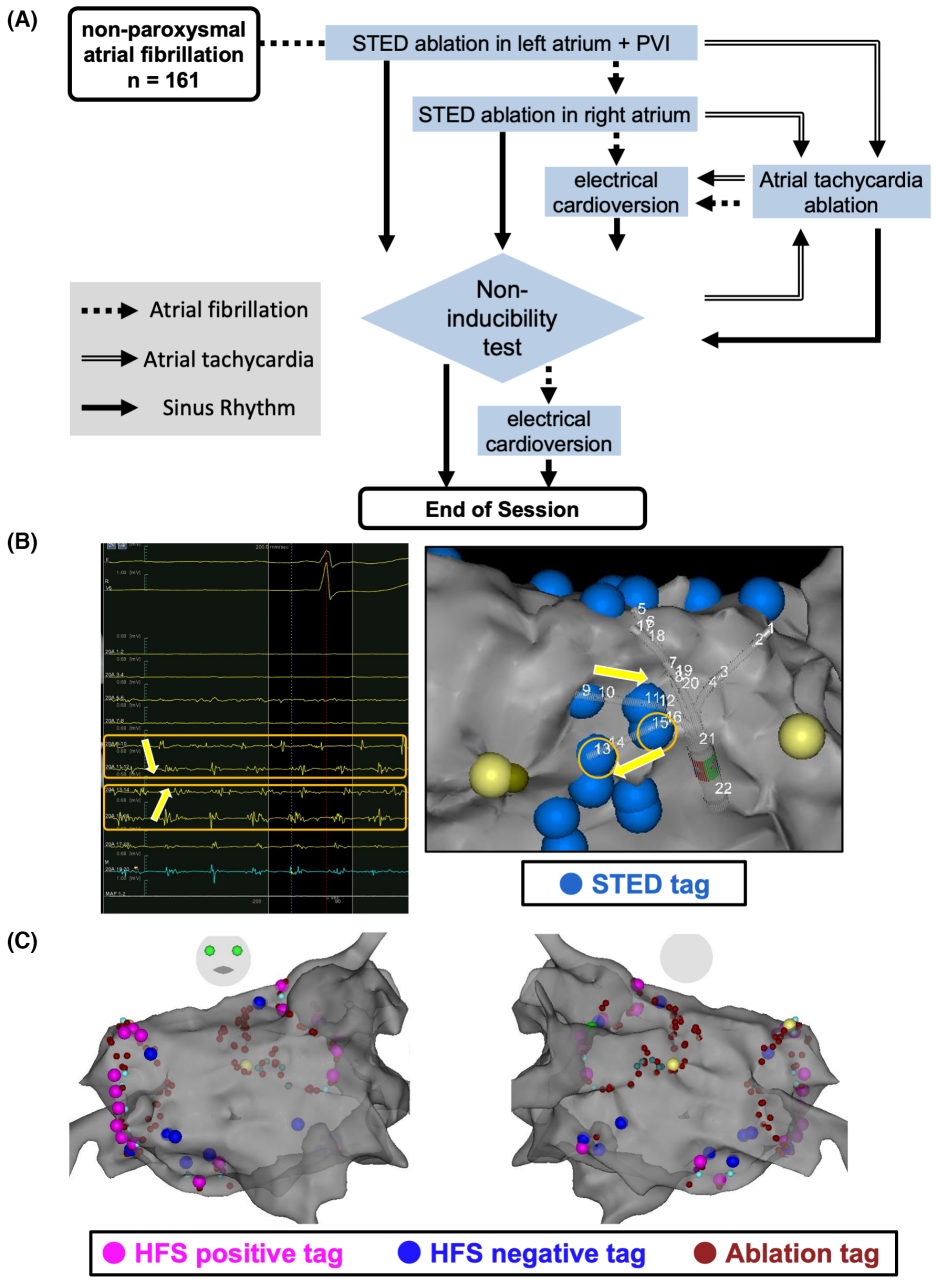
Study protocol. (A) The STED within the entire left and right atria during atrial fibrillation was tagged and ablated. If atrial fibrillation remained unchanged, we applied electrical cardioversion and tested the non‐inducibility before the procedure ended. (B) Identification of STED using a PentaRay catheter. (C) Identification of GP points by HFS and a GP ablation plus antral pulmonary vein isolation. GP, ganglionated plexi; HFS, high‐frequency stimulation; PVI, pulmonary vein isolation; STED, spatiotemporal electrogram dispersion.

The protocol of the STED ablation strategy is presented in Figure 1A. At the same time that the entire LA geometry was constructed using a multielectrode mapping catheter (PentaRay; Biosense Webster, Inc.), simultaneous STED, CFAE, and low‐voltage area (LVA) mapping during AF was conducted with an electroanatomic mapping system (CARTO‐3; Biosense Webster, Inc.). In all cases, the same operator in our center identified STED areas displaying both spatial and temporal dispersion of the intra‐atrial signals recorded for 2.5 s (Figure 1B).^10^ When five atrial cycles of STED could be detected within 2.5 s even if occasionally rather than persistently, the 2.5‐s recording in the same area was obtained repeatedly, and thus the STED area was defined with the confirmation of the reproducibility of STED. In the present study, using CARTO‐3, intra‐atrial bipolar electrograms with low amplitudes (0.03–0.2 mV) and very short intervals (15–80 ms) were defined as CFAE segments and interval confidence levels of ≥40 segments for 2.5 s were defined as CFAE areas.^11^ In terms of the LVA, electrogram amplitudes ≤0.3 mV were defined as low voltages during AF.^12^, ^13^

Radiofrequency (RF) ablation was conducted with a point‐by‐point technique using contact force (CF) and the ablation index showing a lesion‐quality marker. RF energy was applied with 40–45 W on the anterior LA, 30–35 W on the posterior LA, and 25 W near the esophagus. As shown in Figure 1A, the PVI and ablating of the STED areas were performed at the same time. When AF remained unchanged after the PVI and STED ablation in the LA, the STED areas within the right atrium (RA) were subsequently tagged and ablated. When AF terminated during the STED ablation in the LA or RA, we finished the procedure after completing the PVI without ablating the remaining STED areas. If there was sustained AF after the PVI and ablating all STED areas within both the LA and RA, electrical cardioversion was applied before the end of the session. As the acute outcome of the ablation, a non‐inducibility test, that is, atrial rapid pacing in the distal coronary sinus (10 V, pacing with 10 ms decrements from 300 to 200 ms or a 2:1 response, three times) was performed before the procedure ended. If sustained AF changed into atrial tachycardia (AT) during ablation or the non‐inducibility test after ablation‐induced AT, we tried to map, ablate, and eliminate it. When LA enlargement was confirmed by echocardiography and/or the CT images before the procedure, RF ablation was conducted with a remote magnetic navigation (RMN) system (Niobe™ ES; Stereotaxis, Inc.) for a stable and safe catheter contact, which easily manipulates the catheter, reduces radiation exposure and may prevent complications because of catheter manipulation despite clinical outcomes comparable to manual procedures using CF.^14^, ^15^

### 2.3 Follow‐up after ablation

For the long‐term outcome after the ablation, the patients were followed up for 24 months postoperatively by outpatient visits approximately every 3 months with a 12‐lead ECG and 24‐h Holter and/or event ECG monitoring as necessary. With a 3‐month blanking period after the procedures, documented arrhythmia events lasting more than 30 s were defined as recurrences of atrial tachyarrhythmias (ATAs), that is, AF and/or AT. It did not matter whether AADs were used to prevent or terminate the ATAs during the follow‐up.

### 2.4 Validation of the STED ablation

To validate the results of this study with a historical cohort, we investigated the outcomes of 136 previous consecutive non‐PAF patients who underwent a conventional ablation for the first time in our center, that is, ganglionated plexi (GP) ablation plus antral PVI (GPPVI) between July 2015 and September 2017. In the conventional GPPVI, we ablated the GP areas where vagal responses were confirmed by high‐frequency stimulation (20 V, pulse rate 50 ms, and pulse width 10 ms for 5 s) in the GP‐related regions. As shown in Figure 1C, this ablation was performed with a spot‐like method, and the PVI was accomplished using these regions of the GP ablation. Hence, the GPPVI was compared as a conventional ablation strategy, and not an excessive ablation strategy modulating the substrate.

### 2.5 Statistical analysis

Numerical variables are represented as the mean ± SD and compared using Student's *t*‐tests. Categorical variables are represented as the *n* (%) and compared using chi‐square tests or Fisher exact tests. Kaplan–Meier curves were assessed and compared by log‐rank tests. The hazard ratio and their 95% confidence interval were computed by Cox regression models. Any variable with a *p* < .1 in the univariate analysis was included in the multivariate Cox models. A *p* < .05 was considered statistically significant for all tests.

All statistical analyses were conducted with EZR software (Saitama Medical Center, Jichi Medical University),^16^ which is a graphical user interface for R (The R Foundation for Statistical Computing). More precisely, it is a modified version of R commander designed to add statistical functions frequently used in biostatistics.

## 3 RESULTS

### 3.1 Patient characteristics

The characteristics of the 161 patients in the study are given in Table 1. Notably, there were 71 patients (44%) with long‐standing persistent AF. The mean AF cycle length (AFCL) recorded in the coronary sinus before ablation was 161 ms and the mean total number of LA STED tag was 56 points. Because the RMN‐guided procedures were conducted in enlarged LA cases, the mean LA volume and diameter measured by CT and transthoracic echocardiography, respectively, and the total number of LA STED tag were significantly larger in the RMN system‐guided patients than that in manual procedure patients (Table S1). One case could not be analyzed after the procedures because the ablation data were corrupted as a result of hardware trouble. However, this case was not excluded from the study enrollment because the ablation procedure was completed according to our protocol without any trouble. Major complications related to the STED ablation occurred in two patients, that is, one had a cardiac tamponade because of a steam pop during an RF application, and the other had gastroparesis.

**TABLE 1:** Baseline characteristics and procedure data.

	*n* = 161
Male	119 (74)
Age, years	66 ± 10
Long‐standing persistent AF	71 (44)
Body mass index, kg/m^2^	25 ± 4
CHA_2_DS_2_‐VASc score	1.8 ± 1.4
LA volume, mL	176 ± 40
LA diameter, mm	43 ± 5
LV end‐diastolic diameter, mm	47 ± 5
LV ejection fraction, %	56 ± 9
RMN‐system use	95 (59)
Procedure data	
Procedure duration, min	204 ± 49
Total number of RF applications	107 ± 25
Total RF time, min	56 ± 14
Total RF energy, kJ	116 ± 29
Fluoroscopy Time, min	31 ± 16
Radiation dose, mGy	332 ± 303
Pre‐AFCL at coronary sinus, ms	161 ± 22
Total number of LA STED tag	56 ± 20
Complications	2

*Note*: Values are the mean ± SD or *n* (%).

Abbreviations: AF, atrial fibrillation; AFCL, AF cycle length; LA, left atrial; LV, left ventricular; RMN, remote magnetic navigation; RF, radiofrequency; STED, spatiotemporal electrogram dispersion.

### 3.2 Distribution of the STED areas

**FIGURE 2:**
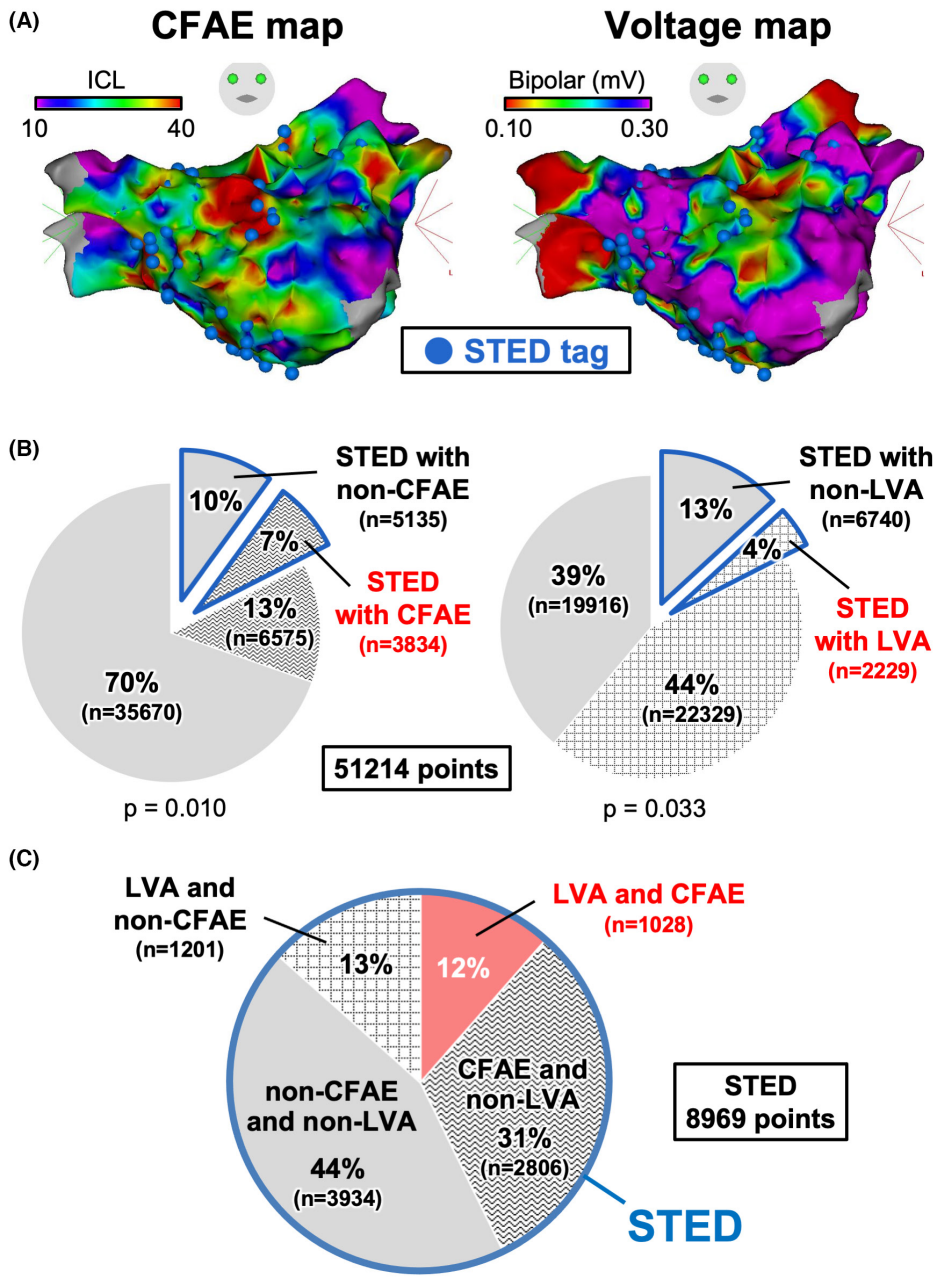
Features of STED areas. (A) Simultaneous mapping of the STED, CFAEs, and LVAs during atrial fibrillation. (B) Ratio of the STED areas, CFAE areas, and LVAs within the entire LA (blue frame: STED area, waveform: CFAE area, dotted‐square: LVA, and filled‐gray: both non‐CFAE areas and non‐LVAs). Ratio of STED areas in CFAE areas (37%) versus in non‐CFAE areas (13%) (*p* = .010); and the ratio of STED areas in LVAs (9%) versus in non‐LVAs (25%) (*p* = .033). (C) Relationship of the STED areas to CFAE areas and LVAs (blue frame: STED area, filled‐pink: both CFAE area and LVA, waveform: both CFAE area and non‐LVA, dotted‐square: both LVA and non‐CFAE area, and filled‐gray: both non‐CFAE area and non‐LVA). CFAE, complex fractionated atrial electrogram; LA, left atrium; LVA, low‐voltage‐area; STED, spatiotemporal electrogram dispersion.

**FIGURE 3:**
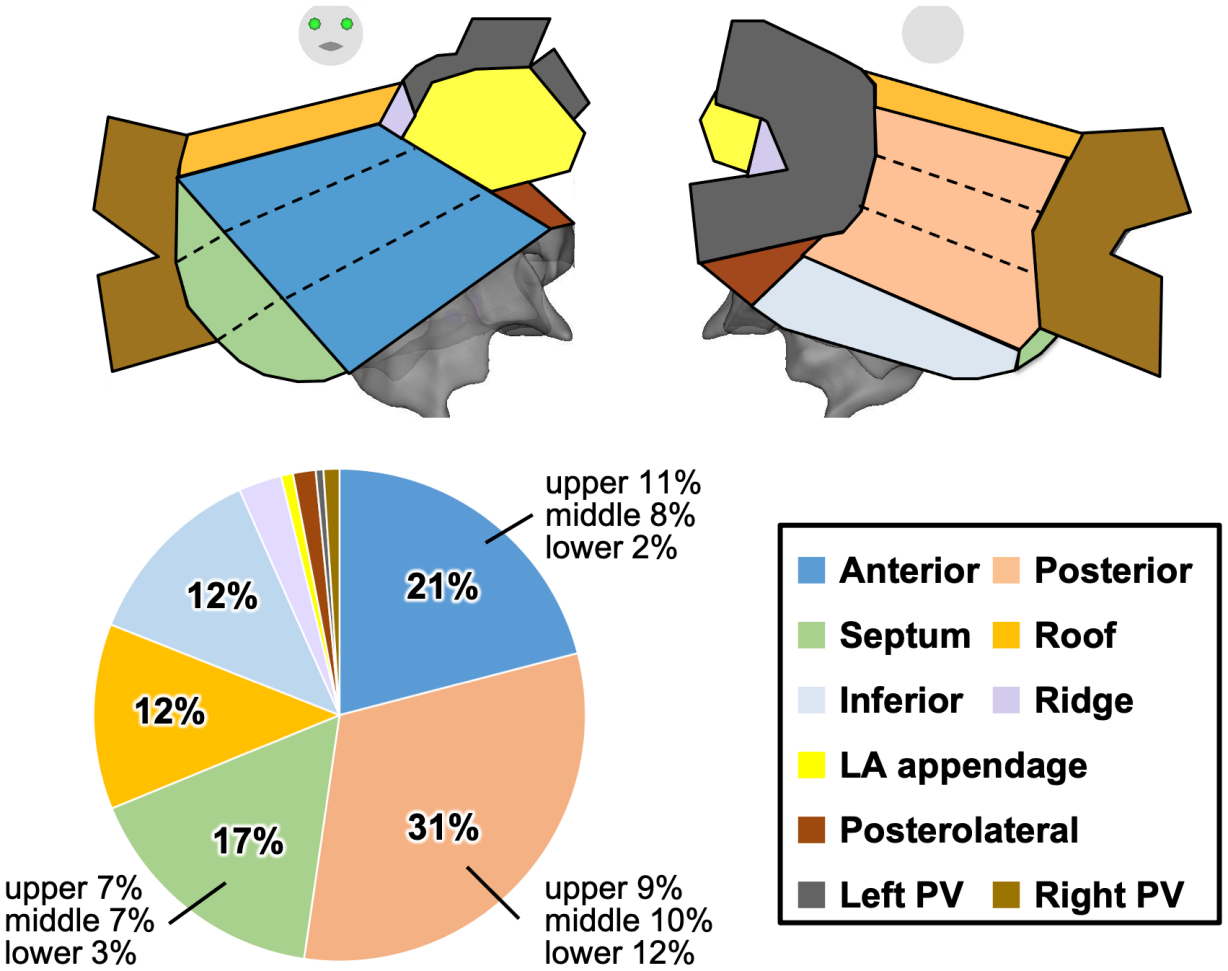
Distribution of STED areas. Distribution of the STED (anterior wall, posterior wall, atrial septum, posterolateral, roof, inferior, PV‐LA appendage ridge, LA appendage, left PV, and right PV). LA, left atrium; PV, pulmonary vein; STED, spatiotemporal electrogram dispersion.

We performed simultaneous mapping of the STED, CFAE, and LVA during sustained AF within the entire LA in 161 consecutive non‐PAF patients without prior procedures (Figure 2A). In this study, we detected and ablated the STED areas within LA and, if necessary, RA. About 110 patients (68%) also underwent the STED ablation of RA. However, STED areas within RA were excluded from the analysis of local potentials because we considered that these potentials might not be obtained well as a result of anatomically poor contact between the five spines of PentaRay and the atrial wall. Among 160 cases, after excluding one case with hardware trouble, 320 ± 82 mapping points were obtained from the entire LA in each case. As shown in Figure 2B, STED areas accounted for 17% of all mapped areas, CFAE areas 20%, and LVAs 48%, and the STED areas were dependent both on whether they were CFAE areas or non‐CFAE areas and on whether they were LVAs or non‐LVAs (*p* = .010 and *p* = .033, respectively). Furthermore, the STED areas with both CFAE areas and LVAs accounted for only 12% of all STED areas, whereas the STED areas with neither CFAE areas nor LVAs accounted for 44% (Figure 2C). Further, we divided the entire LA into 10 regions and counted the distribution of STED points in each region, that is, anterior wall (upper, middle, and lower), posterior wall (upper, middle, and lower), atrial septum (upper, middle, and lower), posterolateral, roof and inferior regions of the LA, PV‐LA appendage ridge, LA appendage, left PV, and right PV.^17^ The STED points were most frequently identified on the lower LA posterior wall and inferior and roof regions of the LA (12%, respectively) (Figure 3).

### 3.3 Outcomes of the STED ablation

**FIGURE 4:**
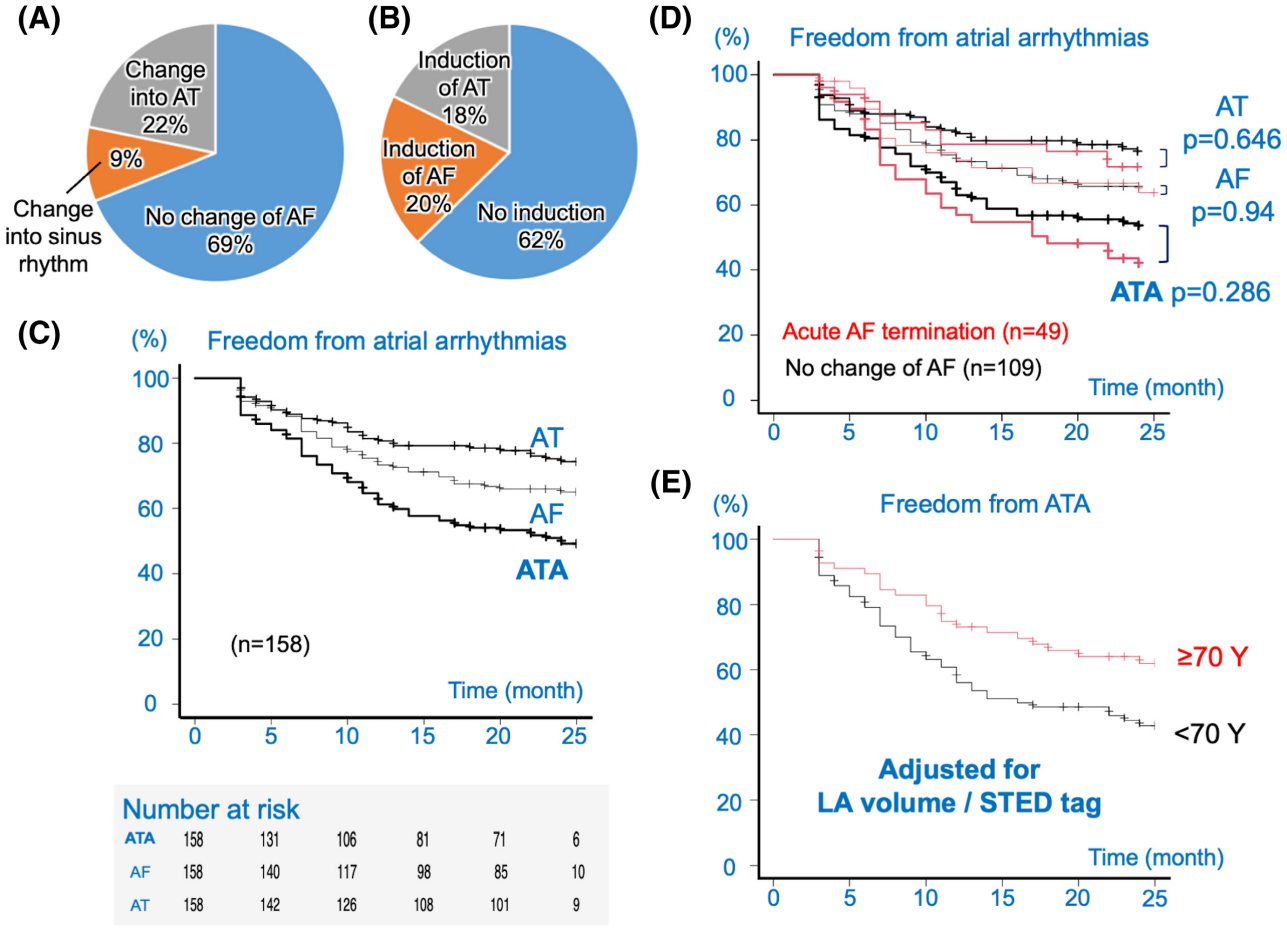
Acute and long‐term outcomes in the STED strategy. (A) Change in AF during the STED ablation. (B) Non‐inducibility after the STED ablation. (C) The black lines indicate the freedom from atrial arrhythmias in the STED strategy (thick line: ATA, thin line: AF, and normal line: AT). (D) The red and black lines indicate the freedom from atrial arrhythmias in the STED strategy with an acute AF termination and the STED strategy with no change of AF (thick line: ATA, thin line: AF, and normal line: AT). (E) Adjusted survival curves after the STED ablation. The red and black lines indicate the freedom from ATAs in patients above and below 70 years, respectively. AF, atrial fibrillation; AT, atrial tachycardia; ATA, atrial tachyarrhythmias; LA, left atrial; STED, spatiotemporal electrogram dispersion; Y, years old.

The change in the sustained AF because of the STED ablation and non‐inducibility of AF/AT just after the STED ablation were evaluated as acute outcomes. As shown in Figure 4A, sustained AF was terminated in 50 of 161 patients (31%) with or without organization into AT. Organization into AT could be achieved in 35 patients (22%), and the AT was terminated in 27 patients (17%). These 35 patients with organized or induced ATs had a total of 72 ATs., that is, 2.1 ± 1.3 ATs per patient; 26 were focal AT or localized reentry, 16—peri‐mitral flutter, 15—common flutter, 2—roof‐dependent macro re‐entry, 1—lower‐loop macro reentry, 2—PV gap‐related, and 10—unknown mechanism. Eventually, 119 patients (74%) required electrical cardioversion to terminate sustained AF/AT. Furthermore, non‐inducibility was obtained in 67 of 107 patients (62%) excluding 54 patients who were unable to undergo inducibility testing because of a prolonged procedure time (Figure 4B). Incidentally, there was no significant difference in the AF termination between the RMN system‐guided patients and manual procedure‐guided patients (30% vs. 33%; *p* = .608).

As long‐term outcomes, the recurrence of ATAs after the first ablation was investigated. Three patients without follow‐up after the 3‐month blanking period were excluded. Regarding AADs, among 158 patients having a follow‐up, 83 had no recurrence of ATAs; 62 with freedom from AAD (75%), 17 with bepridil (21%), 2 with bisoprolol (2%), and 2 with amiodarone (2%). After a follow‐up of 22 ± 6 months, a Kaplan–Meier curve estimated that the ratios of freedom after 24 months from ATAs, AF, and AT were 49%, 65%, and 74%, respectively (Figure 4C). Among them, the ratios of freedom from ATAs, AF, and AT were comparable between the RMN system‐guided patients and manual procedure‐guided patients: 46% versus 55% (*p* = .164); 62% versus 70% (*p* = .173); and 73% versus 77% (*p* = .456) (Figure S1, red line and black line, respectively). Furthermore, the achievement of AF termination as an acute effect did not improve the clinical outcome of the freedom from ATAs, AF, and AT; 41% versus 53% (*p* = .286); 64% versus 66% (*p* = .94); and 72% versus 76% (*p* = .646) (Figure 4D, red line and black line, respectively).

### 3.4 Determinant factors of ATA recurrence

To investigate the determinant factors of the higher recurrence ratio of atrial arrhythmias, a multivariate analysis of the survival curve was performed using a Cox proportional hazards regression. The patient characteristics and ablation data of 158 patients were analyzed (Table 2). The independent determinant of the recurrence of ATAs was only a non‐elderly age, and that of AT was only the total number of tagged and ablated STED points in the LA rather than the age. Therefore, to visually understand the effectiveness of the difference between those above and below 70 years old, the survival curves were adjusted for other determinant factors (Figure 4E). The adjusted survival curves showed that the freedom ratio from ATAs was considerably higher in elderly patients than in non‐elderly patients (62% vs. 43%). On the other hand, the mean AFCL before ablation, the mean number of tagged and ablated LA STED points, AF termination rate as the acute outcome, and the freedom ratio from AT were comparable between elderly and non‐elderly patients despite the higher freedom ratio from ATAs and AF in elderly patients; AFCL was 164 versus 160 ms (*p* = .193); LA STED tag was 58 points versus 55 points (*p* = .428); AF termination rate was 30% versus 32% (*p* = .863); the freedom ratio from AT was 77% versus 73% (*p* = .45); the freedom ratio from ATAs was 61% versus 42% (*p* = .018); the freedom ratio from AF was 74% versus 59% (*p* = .053) (Figure S2, red line and black line, respectively). Further, there was no difference in the monitoring intensity between elderly and non‐elderly patients (21 vs. 22 months, *p* = .813). In short, the STED ablation was more effective for an elderly patient with non‐PAF.

**TABLE 2:** Determinant factors of atrial arrhythmia recurrence.

	ATA recurrence				AF recurrence				AT recurrence	
	Univariate		Multivariate		Univariate		Multivariate		Univariate	
	HR (95% CI)	*p*	HR (95% CI)	*p*	HR (95% CI)	*p*	HR (95% CI)	*p*	HR (95% CI)	*p*
Male	1.07 (0.64–1.79)	.789			1.42 (0.73–2.77)	.302			0.89 (0.44–1.79)	.736
Age	0.98 (0.96–1.00)	.025*	0.98 (0.95–1.00)	.022^†^	0.98 (0.96–1.01)	.136			0.98 (0.95–1.01)	.189
Long‐standing persistent AF	1.41 (0.89–2.21)	.141			1.90 (1.09–3.3)	.024*	1.59 (0.89–2.83)	.119	1.00 (0.52–1.91)	.993
Body mass index	1.00 (0.94–1.07)	.942			1.03 (0.95–1.10)	.485			1.00 (0.92–1.10)	.966
CHA_2_DS_2_‐VASc score	0.90 (0.77–1.07)	.226			0.95 (0.78–1.16)	.597			0.87 (0.69–1.11)	.276
LA volume	1.01 (1.00–1.01)	.022*	1.01 (1.00–1.01)	.073	1.01 (1.00–1.02)	.001*	1.01 (1.00–1.02)	.092	1.01 (1.00–1.01)	.108
LA diameter	1.03 (0.98–1.07)	.239			1.08 (1.02–1.14)	.006*	1.03 (0.96–1.10)	.371	1.02 (0.96–1.09)	.516
LV end‐diastolic diameter	1.01 (0.97–1.05)	.745			1.02 (0.97–1.07)	.462			1.01 (0.95–1.08)	.760
LV ejection fraction	1.00 (0.97–1.02)	.754			1 (0.97–1.03)	.995			0.99 (0.96–1.03)	.716
Pre‐AFCL at coronary sinus	1.00 (0.99–1.01)	.746			0.99 (0.98–1.01)	.322			1.01 (0.99–1.02)	.442
Total number of LA STED tag	1.01 (1.00–1.03)	.037*	1.01 (1.00–1.02)	.147	1.01 (0.99–1.02)	.360			1.02 (1.00–1.03)	.042*
Total RF time	1 (1.00–1)	.556			1 (1.00–1)	.798			1 (1.00–1.00)	.372
RMN‐system use	1.39 (0.86–2.24)	.176			1.49 (0.83–2.66)	.183			1.29 (0.66–2.53)	.462

Abbreviations: AF, atrial fibrillation; AFCL, AF cycle length; AT, atrial tachycardia; ATA, atrial tachyarrhythmias; CI, confidence interval; HR, hazard ratio; LA, left atrial; LV, left ventricular; RF, radiofrequency; RMN, remote magnetic navigation; STED, spatiotemporal electrogram dispersion.

* *p* < .1, Statistical significance in the univariate linear regression analysis.

^†^ *p* < .05, Statistical significance in the multivariate linear regression analysis.

### 3.5 Comparison with the conventional strategy

**FIGURE 5:**
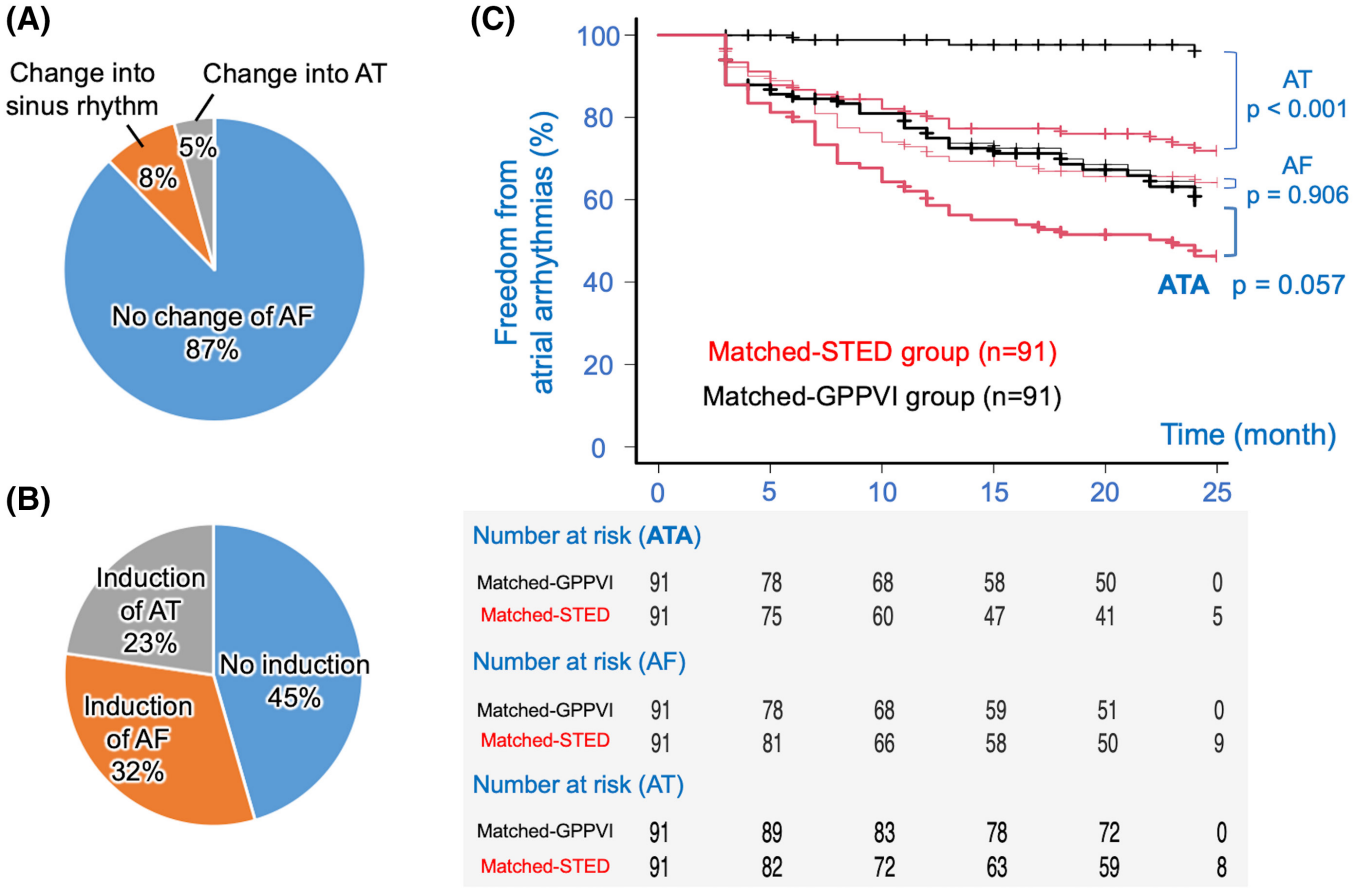
Acute and long‐term outcomes in the conventional strategy and a comparison between the strategies. (A) Change in the AF in the conventional strategy. (B) Non‐inducibility in the conventional strategy. (C) Comparison between the strategies. The red and black lines indicate the freedom from atrial arrhythmias in the matched‐STED group and matched‐GPPVI group patients (thick line: ATA, thin line: AF, and normal line: AT). AF, atrial fibrillation; AT, atrial tachycardia; ATA, atrial tachyarrhythmias; GPPVI, ganglionated plexi ablation plus antral pulmonary vein isolation; STED, spatiotemporal electrogram dispersion.

The outcomes in this study were compared with those of the conventional ablation. In the conventional strategy, nine patients were excluded because of no follow‐up action after the blanking period. Concerning both AF termination and non‐inducibility as acute outcomes, the STED strategy in this study was significantly more effective than the conventional strategy: AF termination ratio was 31% versus 13% (*p* < .001) (Figures 4A and 5A); non‐inducibility ratio was 62% versus 45% (*p* = .036) (Figures 4B and 5B). However, comparing these results with the conventional strategy using an adjustment by a propensity score matching method as given in Table 3, the Kaplan–Meier curves showed that the matched‐STED group had lower freedom from ATAs because of significantly lower freedom from AT than the matched‐GPPVI group, despite a comparable ratio of freedom from AF between the two groups: the freedom ratio from ATAs was 46% versus 59% (*p* = .057); that from AF was 64% versus 61% (*p* = .906); that from AT was 72% versus 95% (*p* < .001) (Figure 5C, red line and black line, respectively). Regarding the determinant factors of the ATA recurrence among the GPPVI group, a multivariate analysis of the Cox proportional hazards regression showed that long‐standing persistent AF rather than age was the only independent determinant (Table S2).

**TABLE 3:** Validation of the STED ablation.

	Matched‐STED group (*n* = 91)	Matched‐GPPVI group (*n* = 91)	*p* value
Male	67 (74)	74 (70)	.74
Age, years	66 ± 11	67 ± 11	.41
Long‐standing persistent AF	45 (50)	40 (44)	.55
Body mass index, kg/m^2^	25 ± 3	24 ± 3	.33
CHA_2_DS_2_‐VASc score	1.9 ± 1.4	2.0 ± 1.4	.71
LA volume, mL	179 ± 37	178 ± 36	.92
LA diameter, mm	44 ± 5	44 ± 6	.80
LV end‐diastolic diameter, mm	48 ± 5	48 ± 5	.68
LV ejection fraction, %	57 ± 9	57 ± 10	.87
RMN‐system use	87 (96)	87 (96)	1
Procedure data			
Procedure duration, min	216 ± 44	245 ± 56	<.01*
Total number of RF applications	106 ± 24	81 ± 21	<.01*
Total RF time, min	56 ± 13	53 ± 12	.11
Total RF energy, kJ	120 ± 28	112 ± 27	.035*
Fluoroscopy time, min	25 ± 10	24 ± 12	.84
Radiation dose, mGy	218 ± 150	233 ± 175	.54

*Note*: Values are the mean ± SD or *n* (%).

Abbreviations: AF, atrial fibrillation; GPPVI, ganglionated plexi ablation plus antral pulmonary vein isolation; LA, left atrial; LV, left ventricular; RF, radiofrequency; RMN, remote magnetic navigation; STED, spatiotemporal electrogram dispersion.

* Statistical significance (*p* < .05).

## 4 DISCUSSION

This study was to validate the effectiveness of the STED ablation targeting rotational activity, which mainly ablated the atrium, beyond the conventional ablation of the atrium. The major findings of our study were as follows: (1) the STED areas depended both on whether having CFAE or not and on whether having low‐voltage or not, (2) despite the more effective acute outcomes, the STED ablation had a significantly higher recurrence of AT than the conventional strategy unlike the comparable recurrence of AF, and (3) the STED ablation targeting rotors was effective for elderly patients with non‐PAF.

### 4.1 Strategy of the non‐PAF ablation

As a possible AF driver, focal activity and/or rotational activity, called rotors have been proposed to play an important role in the mechanism of sustained AF based on previous studies, but the exact mechanism is incompletely understood because the most suitable method for identifying these focal activities and rotors is still controversial.^18^, ^19^ Some previous meta‐analyses reported that ablation for substrate modification plus a PVI has no additional benefit over a PVI alone, while a recent randomized control trial (RCT) implied that an additional substrate ablation added to the PVI might be promising.^4^, ^5^, ^20^ Further, modulating the cardiac autonomic nerve system, which plays a critical role in forming triggers and substrates of AF, and ablating LVAs have been reported as one of the effective ablation strategies for non‐PAF, but the outcomes differ between the study groups.^11^, ^17^, ^21^, ^22^, ^23^, ^24^, ^25^ Alternatively, it has been reported that ablation targeting rotors is an effective strategy for non‐PAF. and the STED, as an indicator of the rotors, is an effective ablation target.^6^, ^7^, ^8^, ^9^, ^10^

### 4.2 The effectiveness of the STED ablation for non‐PAF

The ratio of rotational activity and focal activity composing fibrillatory conduction differs in each case. The strategy of the STED was mainly to target rotational activity. Compared with our conventional strategy, this study did not demonstrate more effective long‐term outcomes despite more effective acute outcomes, which was the same as a recent study about rotor ablation reporting that a focal impulse and rotor modulation‐guided ablation did not affect the clinical outcome despite both AF termination and non‐inducibility.^26^ Surprisingly, only a non‐elderly age was an independent determinant of ATA recurrence after the STED ablation for non‐PAF rather than long‐standing persistent AF or an enlarged LA, which was conventionally considered as a key factor. It has not generally been thought that non‐elderly patients with non‐PAF are more refractory to ablation, and in fact, ATA recurrence after a conventional ablation without modulation of the LA substrate including rotors had no significant correlation with the age. However, a sub‐analysis of the recent RCT above reported the effectiveness of the substrate ablation plus a PVI strategy in elderly patients with non‐PAF.^20^ This result suggests that the aging process might be involved in the mechanism of the atrial substrate generating rotors or its propensity. A previous study showed that an increase in the reentrant substrate is influenced by the aging process, such as fibrosis with myofibroblast proliferation and gap junction coupling or uncoupling.^27^, ^28^ Handa et al. demonstrated using optical mapping of Langendorff‐perfused rat hearts that the degree of gap junction coupling degree in fibrosis influences the fibrillatory mechanism and persistency such as organized and/or disorganized activity,^29^ and thus such a change in the gap junction coupling by aging might bring about organized rotors and make it easier and to identify the STED properly. This could be the main reason for the difference in the STED effectiveness. Moreover, Kapur et al. reported that the presence of an AF family history is a predictor of a poorer freedom from arrhythmias following catheter ablation in persistent AF patients, and Shoemaker et al. reported that higher genetic susceptibility to AF is involved in younger age at ablation.^30^, ^31^ These studies suggest that genetic factors might also complicate the component of fibrillatory conduction in non‐elderly patients.

In addition, the existence of focal activity could be another possible reason for the STED effectiveness, which has also been considered to be one of the hypotheses proposed as the mechanism of AF persistence.^19^, ^32^ Nayyar et al. reported in their study using real‐time mapping in 78 patients that some rotational activities might be passive and not the main mechanism of AF persistency because rotational activities were less common than focal activities and evolved from neighboring focal activity.^33^ Furthermore, Houston et al. reported that the mean perimeter of the rotor core was 1.5 mm, and the rotor size could be less than 1 mm in diameter in their study on optical mapping using fetal bovine myocytes.^34^ The unsatisfactory effectiveness of ablation targeting the STED might be caused by increasing passive rotational activity and miniaturizing rotors, and the existence probability and size of the rotors might become altered depending on the differences in the above‐mentioned substrate and the complexity of the fibrillatory conduction because of the aging process or genetic factors. This might explain the reason why STED ablation was more effective in elderly patients. This study suggested that in elderly patients, the main mechanism of AF persistency resulted from rotors that were active rotational activities, whereas in non‐elderly patients, the mechanism AF persistency resulted from other activities composing the fibrillatory conduction such as focal activity rather than repetitive rotational activities containing rotors identified by the STED. To sum it up, the main mechanism of AF persistency might change as one gets older. Furthermore, it is popularly believed that the complexity of the fibrillatory conduction is associated with atrial structural and electrical remodeling, which is usually confirmed by the AFCL during ablation. However, the present study showed that conventional structural remodeling‐related factors and the global AFCL in the coronary sinus were less associated with AF recurrence than the age and duration of AF persistency. This suggested that global AFCL does not really reflect the complexity of the fibrillatory conduction because the AFCL measurement sites might be located far from the AF driver sites, and thus controlling the fibrillatory conduction needs treatments based on local activity rather than global measurements. Of course, there could be other underlying possibilities, mechanisms, and reasons for the difference in the STED effectiveness. Since various arrhythmia imaging systems and substrate evaluating methods have been recently developed, future studies are needed to confirm the local component of the fibrillatory conduction and uncover the mechanism of AF persistency.

### 4.3 Strategy of rotor modulation

In this study, the STED ablation as a rotor ablation was more effective for the acute outcomes than the conventional strategy. However, the STED ablation resulted in a higher recurrence of all ATAs including AT caused by extensive LA ablation. Moreover, the increase in STED ablation points was the determinant factor causing post‐ablation ATs. This result is consistent with the common recognition that more extensive ablation of the LA increases iatrogenic ATs.^35^ A previous study reported that ganglionated plexus ablation with non‐excessive ablation of the LA is superior to linear ablation ablating the LA widely, regarding the freedom from both AF and AT.^36^ In this study, the collective ablation method in the STED strategy, unlike the spot‐like method in conventional the GPPVI strategy, might have a disadvantage of excessive ablation, which is greater than the advantage of rotor modification even though STED completely reflected rotors. This present study could not clarify the optimal ablation strategy for non‐PAF, and whether we should ablate more points to cover the rotors fully and connect the STED areas to anatomical block lines or fewer points to prevent extensive LA ablation. However, at least in the case of non‐elderly patients with a lesser reentrant substrate associated with aging, it might be hard to eliminate the fibrillatory conduction by the STED ablation targeting repetitive rotational activities containing rotors, and thus non‐excessive ablation of the atrium should be considered to avoid post‐ablation ATs. For example, it is necessary to carefully select cases where the advantages of the extensive LA ablation may outweigh the disadvantages, such as redo procedure cases where a PVI has already been performed. We might not have to extensively ablate all identified rotational activities in the first session.

### 4.4 Study limitation

We had some limitations in this study. First, this study was not a multicenter study, but we could show the consistency of the STED identification because of a single‐center study. Secondly, this design was not an RCT. However, we consecutively enrolled the subjects and analyzed the data with a propensity score matching method for validation. Thirdly, the historical cohort in this study was not a standard circumferential PVI. However, this was very useful for the validation of STED ablation and comparison to “non‐excessive” ablation since this strategy had significantly lower total RF energy and the total number of RF applications than the STED ablation strategy. Next, the inducibility testing after ablation could not be performed in one‐third of the population because the procedure duration was extended. Lastly, it was difficult to confirm active rotational activities as AF drivers even though rotational activities can be identified by STED as mentioned above. Future work is required for these limitations.

## 5 CONCLUSIONS

The STED ablation targeting rotors is effective for elderly patients with non‐PAF, and thus the main mechanism of AF persistency and the component of fibrillatory conduction might be different between elderly and non‐elderly patients. However, it is necessary to pay attention to the disadvantage, that is post‐ablation AT, with the STED ablation, which might eliminate the fibrillatory conduction and control the AF drivers in the atrium.

## CONFLICT OF INTEREST STATEMENT

The authors have no financial conflicts of interest to disclose concerning the manuscript.

## DECLARATIONS

*Approval of the research protocol*: Approved by the ethics committee in Takatsuki General Hospital on December 23, 2019. The approval number is 2019‐72. *Informed consent*: Obtained from all patients. *Registry and Registration No. the study*: N/A. *Animal studies*: N/A.

## Supporting information


Data S1.
