# Supplementary figures and images for: The spatiotemporal electrogram dispersion ablation targeting rotors is more effective for elderly patients than non‐elderly population
Source: J Arrhythm. 2023 May 3;39(3):315–26. doi: 10.1002/joa3.12860 (PMC10264740; doi:10.1002/joa3.12860)

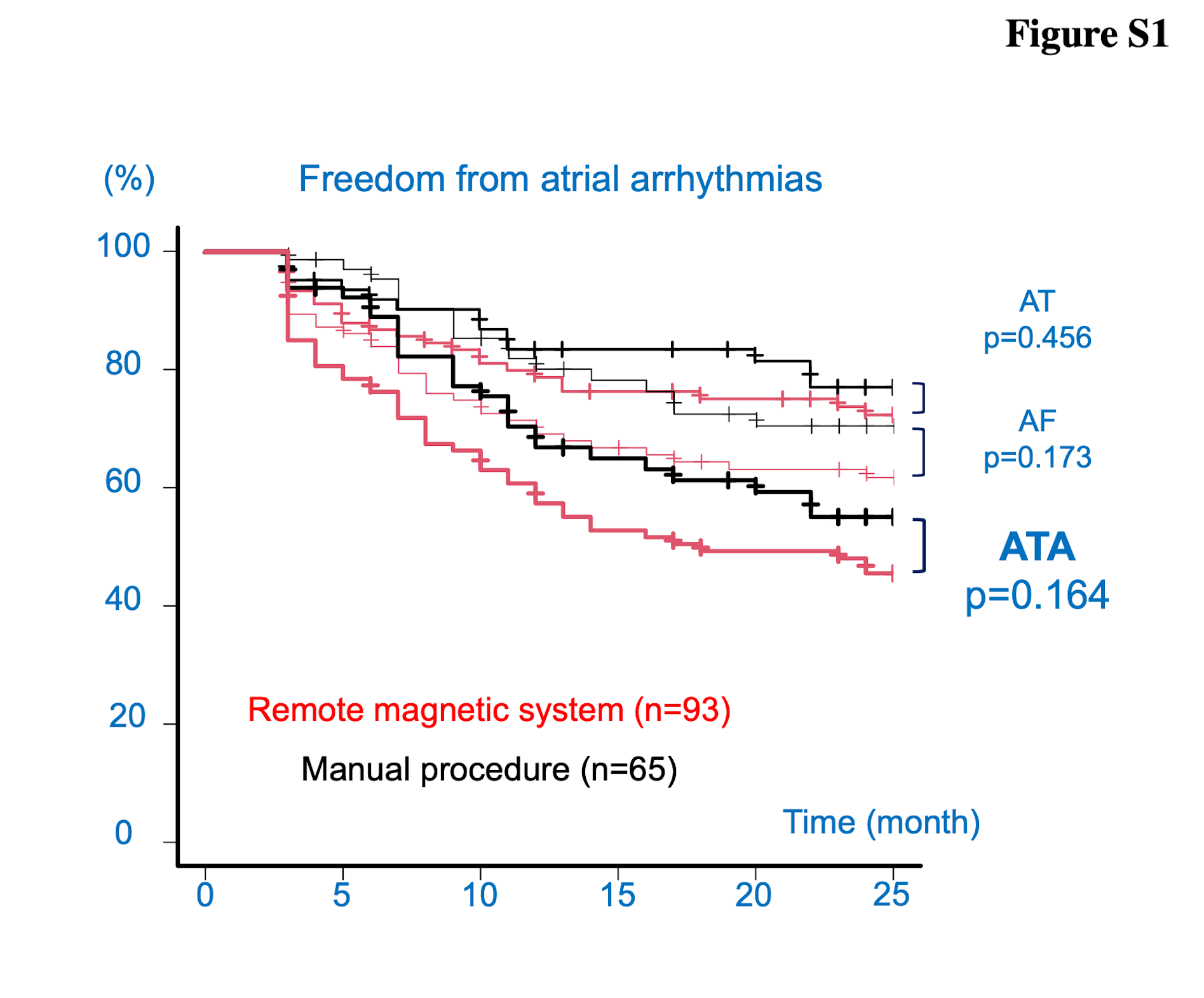

Supplement: Supplementary file 1 — Data S1. [file JOA3-39-315-s001.zip › joa312860-sup-0002-FigureS1.tiff]

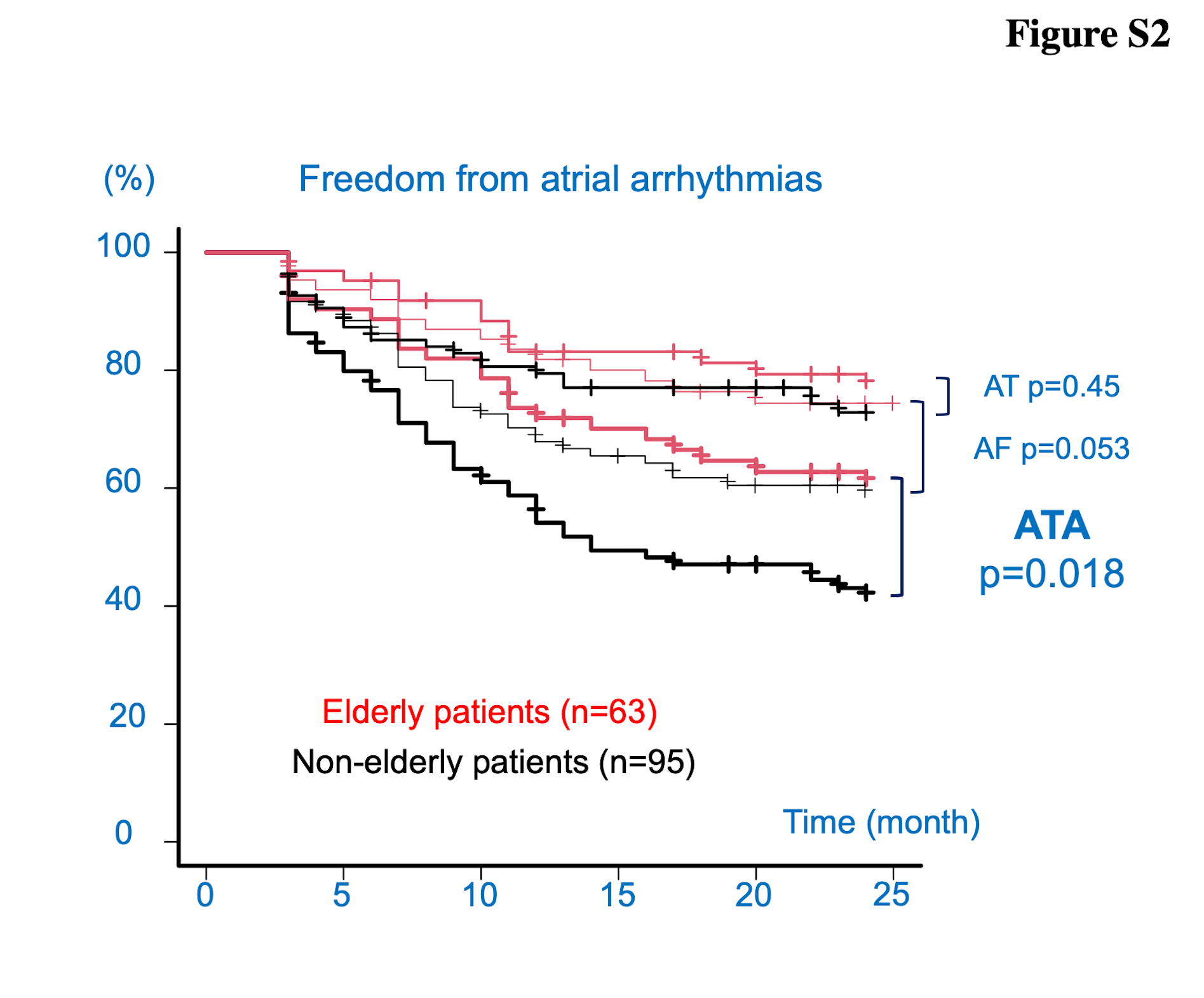

Supplement: Supplementary file 1 — Data S1. [file JOA3-39-315-s001.zip › joa312860-sup-0003-FigureS2.tiff]
