# Supplementary material for: The spatiotemporal electrogram dispersion ablation targeting rotors is more effective for elderly patients than non‐elderly population
Source: J Arrhythm. 2023 May 3;39(3):315–26. doi: 10.1002/joa3.12860 (PMC10264740; doi:10.1002/joa3.12860)
Supplement: Supplementary file 1 — Data S1. [file JOA3-39-315-s001.zip › joa312860-sup-0001-Supinfo.doc]

**Supporting Information**

**Online Supplement Data 1.	Table S1**

**Online Supplement Data 2.	Figure S1**

**Online Supplement Data 3.	Figure S2**

**Online Supplement Data 4.	Table S2**

**Table**

**Table S1. Consecutive patient characteristics and procedure data in the RMN system and manual procedure**

| **Consecutive** | **RMN (n=95)** | **Manual (n=66)** | **p value** |
| --- | --- | --- | --- |
| Male | 71 (75) | 48 (73) | 0.86 |
| Age, Y | 66±10 | 66±10 | 0.97 |
| Long-standing persistent AF | 49 (52) | 22 (33) | 0.03* |
| Body Mass Index, kg/m^2^ | 25±4 | 25±4 | 0.35 |
| CHA_2_DS_2_-VASc score | 1.9±1.4 | 1.8±1.4 | 0.88 |
| LA volume, ml | 186±43 | 161±30 | <0.01* |
| LA diameter, mm | 44±5 | 42±5 | <0.01* |
| LV end-diastolic diameter, mm | 48±5 | 46±6 | 0.08 |
| LV ejection fraction, % | 57±8 | 54±9 | 0.03* |
| Procedure data |  |  |  |
| Procedure duration, min | 214±44 | 190±52 | <0.01* |
| Total number of RF applications | 107±24 | 106±27 | 0.72 |
| Total RF time, min | 57±13 | 55±15 | 0.42 |
| Total RF energy, kJ | 122±27 | 109±31 | <0.01* |
| Fluoroscopy Time, min | 23±7 | 42±20 | <0.01* |
| Radiation dose, mGy | 219±124 | 495±398 | <0.01* |
| Pre-AFCL at coronary sinus, ms | 162±23 | 161±20 | 0.79 |
| Total number of LA STED tag | 60±22 | 51±16 | <0.01* |
| Complications | 1 | 1 |  |

Values are the mean±S.D. or n (%).

*Statistical significance (p>0.05).

AF = atrial fibrillation; LA = left atrial; LV = left ventricular; RF = radiofrequency; RMN = remote magnetic navigation.

**Table S2. Determinant factors of atrial arrhythmias recurrence in the conventional strategy**

|  | **ATA recurrence** | | | | **AF recurrence** | | | | **AT recurrence** | |
| --- | --- | --- | --- | --- | --- | --- | --- | --- | --- | --- |
|  | **Univariate** | | **Multivariate** | | **Univariate** | | **Multivariate** | | **Univariate** | |
|  | **HR (95% CI)** | **p** | **HR (95% CI)** | **p** | **HR (95% CI)** | **p** | **HR (95% CI)** | **p** | **HR (95% CI)** | **p** |
| Male | 0.84  (0.46-1.52) | 0.556 |  |  | 0.78  (0.42-1.46) | 0.439 |  |  | 0.88  (0.22-3.51) | 0.853 |
| Age | 0.98  (0.96-1.01) | 0.124 |  |  | 0.98  (0.96-1.01) | 0.144 |  |  | 1.01  (0.94-1.08) | 0.837 |
| Long-standing persistent AF | 1.93  (1.10-3.38) | 0.022* | 1.80  (1.02-3.18) | 0.044† | 1.85  (1.02-3.34) | 0.042* | 1.73  (0.95-3.14) | 0.073 | 2.56  (0.64-10.24) | 0.184 |
| Body Mass Index | 1.06  (0.98-1.15) | 0.148 |  |  | 1.06  (0.98-1.16) | 0.162 |  |  | 1.09  (0.91-1.30) | 0.343 |
| CHA_2_DS_2_-VASc score | 0.94  (0.76-1.16) | 0.547 |  |  | 0.95  (0.77-1.19) | 0.674 |  |  | 1.00  (0.62-1.62) | 0.994 |
| LA volume | 1.01  (1-1.02) | 0.050* | 1.01  (1.00-1.01) | 0.110 | 1.01  (1-1.02) | 0.047* | 1.01  (1.00-1.02) | 0.088 | 1.01  (1.00-1.03) | 0.123 |
| LA diameter | 1.01  (0.96-1.06) | 0.680 |  |  | 1.01  (0.96-1.07) | 0.686 |  |  | 1.09  (0.97-1.22) | 0.158 |
| LV end-diastolic diameter | 1.04  (0.98-1.10) | 0.205 |  |  | 1.04  (0.98-1.10) | 0.172 |  |  | 1.04  (0.91-1.19) | 0.544 |
| LV ejection fraction | 0.99  (0.96-1.02) | 0.407 |  |  | 0.99  (0.96-1.02) | 0.454 |  |  | 0.99  (0.93-1.05) | 0.657 |
| Total RF time | 1  (1.00-1) | 0.712 |  |  | 1  (1.00-1) | 0.568 |  |  | 1.00  (1.00-1.00) | 0.803 |
| RMN-system use | 1.86  (0.26-13.46) | 0.540 |  |  | 1.63  (0.22-11.86) | 0.628 |  |  | -  (0- ∞) | 0.999 |

*p<0.1, Statistical significance in the univariate linear regression analysis. †p<0.05, Statistical significance in the multivariate linear regression analysis.

ATA = atrial tachyarrhythmias; AF = atrial fibrillation; AT = atrial tachycardia; HR = hazard ratio; CI = confidence interval; LA = left atrial; LV = left ventricular; RF = radiofrequency; RMN = remote magnetic navigation.

**Figure Legends**

**Figure S1. Long-term outcomes in the STED strategy with the RMN system and with manual procedure**

The red and black lines indicate the freedom from atrial arrhythmias in the STED strategy with the RMN system and the STED strategy with manual procedure (thick line: ATA, thin line: AF, and normal line: AT).

STED = spatiotemporal electrogram dispersion; RMN = remote magnetic navigation; ATA = atrial tachyarrhythmias; AF = atrial fibrillation; AT = atrial tachycardia.

**Figure S2. Long-term outcomes in elderly and non-elderly patients with the STED strategy**

The red and black lines indicate the freedom from atrial arrhythmias in elderly and non-elderly patients with the STED strategy (thick line: ATA, thin line: AF, and normal line: AT).

STED = spatiotemporal electrogram dispersion; ATA = atrial tachyarrhythmias; AF = atrial fibrillation; AT = atrial tachycardia.
